# The Dengue Vector *Aedes aegypti* Contains a Functional High Mobility Group Box 1 (HMGB1) Protein with a Unique Regulatory C-Terminus

**DOI:** 10.1371/journal.pone.0040192

**Published:** 2012-07-03

**Authors:** Fabio Schneider Ribeiro, Isabel Caetano de Abreu da Silva, Vitor Coutinho Carneiro, Fabrício dos Santos Belgrano, Ronaldo Mohana-Borges, Ivone de Andrade Rosa, Marlene Benchimol, Nathalia Rocha Quintino Souza, Rafael Dias Mesquita, Marcos Henrique Ferreira Sorgine, Felipe Gazos-Lopes, Amanda Roberta Revoredo Vicentino, Wenjie Wu, Renata de Moraes Maciel, Mario Alberto Cardoso da Silva-Neto, Marcelo Rosado Fantappié

**Affiliations:** 1 Programa de Biologia Molecular e Biotecnologia, Instituto de Bioquímica Médica, Universidade Federal do Rio de Janeiro, Rio de Janeiro, Brasil; 2 Instituto de Biofísica Carlos Chagas Filho, Universidade Federal do Rio de Janeiro, Rio de Janeiro, Brasil; 3 Departamento de Bioquímica, Instituto de Química, Universidade Federal do Rio de Janeiro, Rio de Janeiro, Brasil; 4 Instituto Nacional de Ciência e Tecnologia em Entomologia Molecular, Universidade Federal do Rio de Janeiro, Rio de Janeiro, Brasil; 5 Universidade Santa Úrsula, Rio de Janeiro, Brasil; 6 Department of Urology, Roswell Park Cancer Institute, Buffalo, New York, United States of America; Centro de Pesquisas René Rachou, Brazil

## Abstract

The mosquito *Aedes aegypti* can spread the dengue, chikungunya and yellow fever viruses. Thus, the search for key molecules involved in the mosquito survival represents today a promising vector control strategy. High Mobility Group Box (HMGB) proteins are essential nuclear factors that maintain the high-order structure of chromatin, keeping eukaryotic cells viable. Outside the nucleus, secreted HMGB proteins could alert the innate immune system to foreign antigens and trigger the initiation of host defenses. In this work, we cloned and functionally characterized the HMGB1 protein from *Aedes aegypti* (AaHMGB1). The AaHMGB1 protein typically consists of two HMG-box DNA binding domains and an acidic C-terminus. Interestingly, AaHMGB1 contains a unique alanine/glutamine-rich (AQ-rich) C-terminal region that seems to be exclusive of dipteran HMGB proteins. AaHMGB1 is localized to the cell nucleus, mainly associated with heterochromatin. Circular dichroism analyses of AaHMGB1 or the C-terminal truncated proteins revealed α-helical structures. We showed that AaHMGB1 can effectively bind and change the topology of DNA, and that the AQ-rich and the C-terminal acidic regions can modulate its ability to promote DNA supercoiling, as well as its preference to bind supercoiled DNA. AaHMGB1 is phosphorylated by PKA and PKC, but not by CK2. Importantly, phosphorylation of AaHMGB1 by PKA or PKC completely abolishes its DNA bending activity. Thus, our study shows that a functional HMGB1 protein occurs in *Aedes aegypt* and we provide the first description of a HMGB1 protein containing an AQ-rich regulatory C-terminus.

## Introduction

It is mandatory to eukaryotic cells to condense and organize their genomic DNA in a supramolecular nucleoprotein structure called chromatin, a conserved structural polymer of DNA and histones whose basic unit is the nucleosome [1]. Dynamic changes in the local or global organization of chromatin are required to perform nuclear activities, including DNA replication, transcription and repair [2], [3]. Maintenance of such a dynamic structure in terms of proper nucleosome distribution and reorganization during nuclear activities is considered crucial to cell survival. Recently, it has been shown that mammalian cells lacking a class of nonhistone proteins, the High Mobility Group Box 1 (HMGB1), contained a substantially reduced amount of histones and nucleosomes [1].

The HMGB proteins are a highly abundant class of nonhistone chromosomal proteins found in all eukaryotic organisms. HMGBs play biologically fundamental roles including DNA transcription, replication, repair and recombination [3], [4], [5]. These proteins act as DNA chaperones, which facilitate assembly of nucleoprotein higher-order chromatin structures by bending or looping DNA or by stabilizing underwound DNA [6]. It is known that HMGB1 proteins regulate transcription by sharply bending DNA to facilitate assembly of other transcription factors to interact and function. Transcription factors also require HMGB1 proteins for high affinity binding to their cognate DNA response elements. Examples of transcription factors that interact with HMGB1 and enhance transcription are the steroid receptors [7], [8], NF-kB/Rel [9], p53 [10], among others.

In addition to its nuclear function, HMGB1 proteins can also act as a pro-inflammatory cytokine upon release into the extracellular environment by necrotic, inflammatory and apoptotic cells [11], [12]. The shuttling of HMGB1 from the nucleus to the cytoplasm and its secretion are triggered by post-translation modifications, mainly acetylation and phosphorylation [13], [14].

Typically, proteins belonging to the HMGB family contain two highly positive (lysine-rich) HMG-box motifs (named HMG-boxes A and B) and a C-terminus rich in aspartic and glutamic acid (named acidic tail) [5]. Although the HMG- boxes are the platforms that recognize and bind DNA, it is now well established that the acidic tails of HMGB1 proteins regulate the binding of the two HMG-boxes to DNA of different topologies (linear, supercoiled, etc) [5], [15], [16], [17].

HMGB proteins have been functionally characterized in organisms ranging from protozoa [18], [19], moss [20], worms [21], plants [22], shrimp [23], insects [24] to mammalian [5]. The HMGB proteins of the fruit fly *Drosophila melanogaster* [25], [26] and the midge *Chironomus tetans* [15], [24], [26] are the best characterized members of this group of proteins in insects [24]. Dorsal switch protein 1 (Dsp1) is the only insect orthologue of mammalian HMGB1 protein found in *Drosophila* [27]. Dsp1 protein was first identified and characterized as a corepressor of the Dorsal protein (Dorsal is a member of the Rel/NF-kB family of transcriptional activators) in *Drosophila* [27]. Subsequent work has established multiple roles of Dsp1 in *Drosophila* embryo development, differentiation and segmentation [28], [29], [30].

To date, no HMGB proteins have been characterized in insect vectors. Here, we have searched databases for HMGB proteins encoded by the Dengue vector *Aedes aegypti.* Our survey yielded one candidate that shared structural features with mammalian HMGB1 and *Drosophila* Dsp1 proteins. Analysis of the *A. aegypti* HMGB1 (AaHMGB1) protein revealed structural and functional similarities, as well as important differences compared to HMGB proteins from other organisms. In this regard, this is the first description of a regulatory AQ-rich domain located just adjacent to the acidic tail in HMGB1 proteins.

## Results

### AaHMGB1 cDNA Cloning and Sequence Analysis

**Figure 1:**
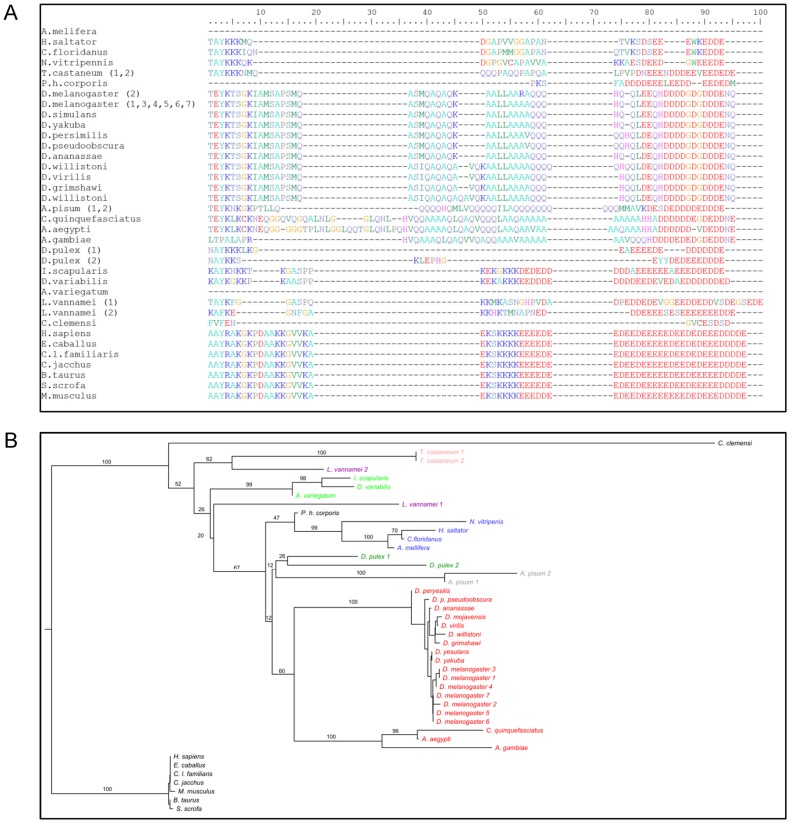
Phylogenetic and sequence analysis of insect HMGB proteins. (A) Phylogenetic tree of HMGB proteins from insects. The unrooted tree was built using the neighbor-joining method based on the alignment of HMGB amino acid sequences. Numbers indicate the bootstrap percentage support (10,000 replicates). (B) Alignment of the C-terminal portion of HMGB proteins from insects. The AQ-rich regions of the insect HMGB proteins run from positions 37 to 81. For comparison purposes, mammalian HMGB1 proteins were also included in the phylogeny analysis and alignment. The different Orders are represented by the different colors.

We searched the *A. aegypti* database (www.vectorbase.org) and identified a cDNA encoding a HMGB1 protein (vectorbase gene: AAEL011380). The *A. aegypti* HMGB1 (AaHMGB1) full length cDNA has an open reading frame of 834 bp, encoding a protein of 278 amino acids with a calculated molecular weight of 31.5 kDa and an isoeletric point of 8.2. Domain analysis showed that AaHMGB1 contains the three part-domain organization typical of the HMGB family, consisting of two HMG-box motifs, A and B, and a C-terminal acidic tail (Figure S1). Curiously, AaHMGB1 protein contains a unique alanine/glutamine-rich (AQ-rich) region within its C-terminal region (Figure S1 and Figure 1A). Comparison of the deduced amino acid sequences of *A. aegypti*, human and *Drosophila* HMGB1 (known as Dsp1) proteins reveals a predictable high similarity within the HMG-boxes (Figure S1). However, comparison of the C-terminal regions revealed striking differences between the human HMGB1 and the dipteran proteins (Figure 1A). Firstly, AaHMGB1 and Dsp1 contain a smaller acidic tail, with only eleven acidic (DE) residues. The stretch of 21 residues of alanine and glutamine (AQ) in AaHMGB1 is located just before and adjacent to its short acidic tail (Figure 1A). Phylogenetic analysis indicated that the acquisition of the AQ-rich region is a peculiarity of Diptera (Figure 1B and Table S1), not found in any other order analyzed (Table S1). By the same token, it is worth to mention that in spite of the AQ-rich domain be found only in dipterans, not all dipterans contain such a domain (which is the case of *Drosophila* HMGD, accession # NM_166480.1 and *Chiromonus* cHMGB1a/b, accession # M93253.1; see Table S1). Moreover, the phylogenetic tree revealed genetic distances among dipterans (compare *Drosophila* with mosquitoes, Figure 1B), which is likely due to the presence of a long glutamine-rich (Q-rich) domain at the N-terminal portion of all *Drosophila* HMGB proteins. This N-terminal Q-rich domain is not present in *Aedes, Culex* or *Anopheles* HMGBs.

### Developmental Expression of AaHMGB1

**Figure 2:**
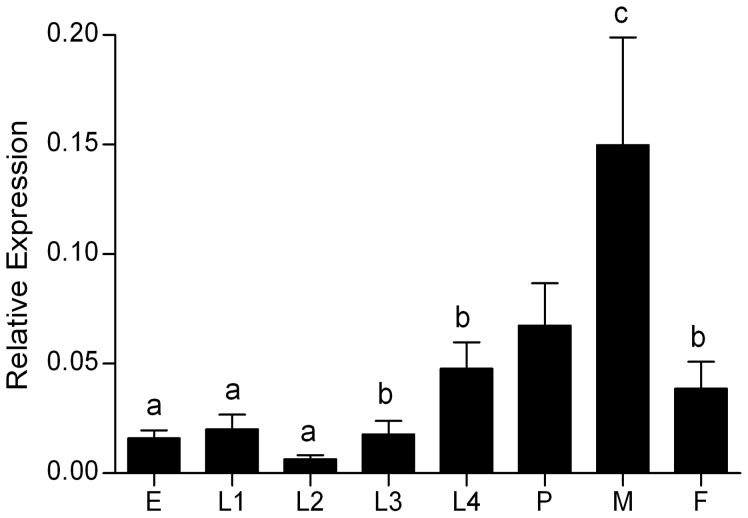
Expression of AaHMGB1 during the different stages of mosquito development. AaHMGB1 mRNA expression was determined by quantitative Real-Time RT-PCR. Eggs (E), 1^st^ instar larvae (L1), 2^nd^ instar larvae (L2), 3^rd^ instar larvae (L3), 4^th^ instar larvae (L4), pupae (P), male (M) and female (F). Values are means of triplicate samples. Columns with different letters are significantly different from each other (a×c (P<0.01), b×c (P<0.05).

Considering the fundamental biological roles that HMGB proteins play in all cell types, and knowing of its cellular abundance, we hypothesized that AaHMGB1 would be expressed in all stages and sexes of the mosquito. Indeed, quantitative real-time reverse transcription PCR analysis (qRT-PCR) showed that AaHMGB1 transcripts are expressed from eggs to adult male and female mosquitoes (Figure 2). The expression of AaHMGB1 was consistently found increased in late stages of the mosquito, reaching its maximum expression in male mosquitoes. Although this finding deserves further investigation, one could anticipate that the high expression of AaHMGB1 in males could be attributed to processes related to the mosquito innate immunity (among other cellular processes), since it has been often observed that immune-related genes are expressed at higher levels in male mosquitoes (unpublished data).

### Cellular Localization of AaHMGB1

**Figure 3:**
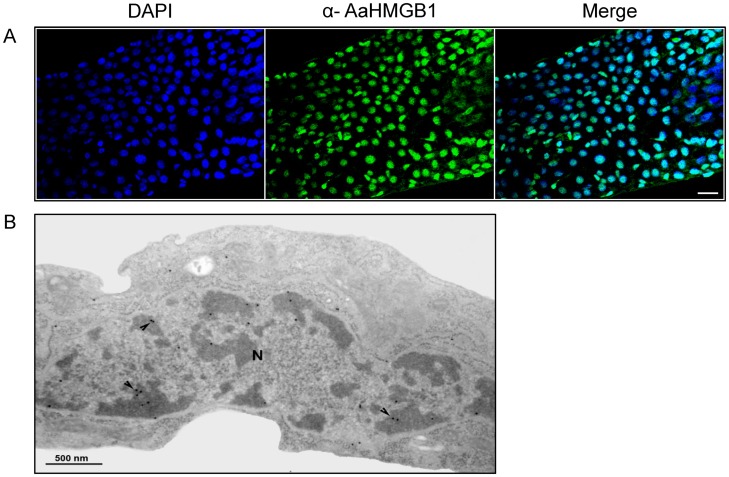
Cellular localization of native AaHMGB1 protein. (A) Immunostaining of AaHMGB1 protein in the midguts of adult sugar-fed mosquitoes. Nuclei were stained with DAPI. AaHMGB1-polyclonal antibody (α-AaHMGB1) was used to detect the endogenous protein; Scale bar: 20 µm. (B) Transmission Electron Microscopy (TEM) of C6/36 mosquito cells using α-AaHMGB1, showing the nucleus (N). The immunogold markers show labeling of AaHMGB1 (arrows) mainly in heterochromatin regions (darker regions of the nuclei). This image is a representative of several cells observed under TEM.

HMGB1 proteins are located in the nucleus of most eukaryotic cells. In surprising to its intranuclear role, HMGB1 can also be secreted by certain cells, and play important roles in inflammation [11], [12], [31]. By performing *in silico* analysis (http://www.cbs.dtu.dk/services/EPipe) we were able to identify a putative nuclear localization signal in AaHMGB1, from position 121 to 151 (Figure S1). In order to establish the cellular location of native AaHMGB1, we performed immune-histochemistry on mosquitós midguts, as well as transmission electron microscopy on C6/36 mosquito cells. We showed that AaHMGB1 localized exclusively in the nuclei of midgut cells (Figure 3A). In addition, the high-powered electron microscopy allowed us to identify AaHMGB1 mainly within heterochromatin regions of mosquito cells (Figure 3B, arrows).

### DNA Transactions by Recombinant AaHMGB1 Proteins

**Figure 4:**
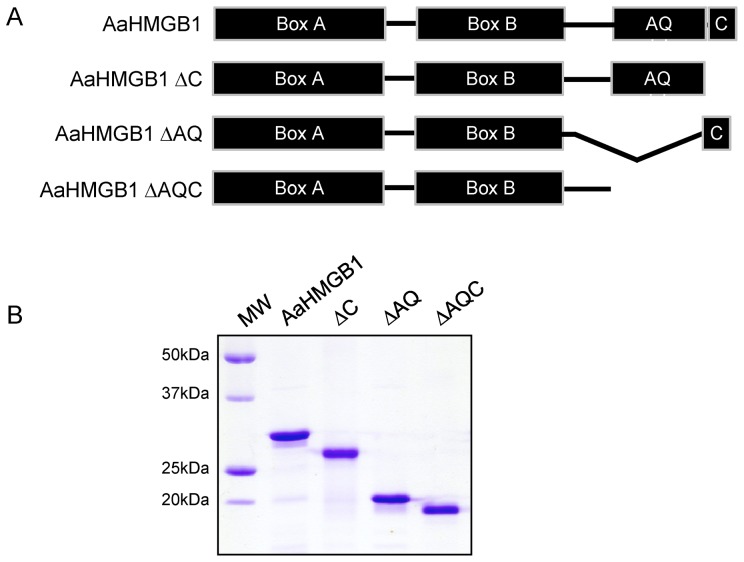
Schematic diagram and SDS-PAGE of the recombinant AaHMGB1 proteins used in this study. (A) Diagram of the recombinant 6×-his-tagged proteins: AaHMGB1, consists of two DNA-binding domains, the HMG box A and HMG box B, a alanine/glutamine-rich (AQ-rich) domain and a short acidic C-terminal domain; AaHMGB1-ΔC lacks only the short acidic C-terminal domain; AaHMGB1-ΔAQ lacks only the AQ-rich domain; AaHMGB1-ΔAQC lacks the entire C-terminus, including the AQ-rich and the short acidic C-terminal domains. (B) SDS-PAGE of the purified recombinant proteins. One microgram of each construct was loaded and analyzed on a 12% SDS-PAGE gel.

As mentioned previously, the acidic tails of HMGB1 proteins modulate the interactions of the HMG-boxes with DNA. In order to test the effect, not only of the acidic tail, but also of the unique AQ-rich region of AaHMGB1, on DNA transactions, we generated three deletion mutants (Figure 4A), as follows: AaHMGB1-ΔC (lacking the short acidic tail); AaHMGB1-ΔAQ (lacking just the AQ-rich region); and AaHMGB1-ΔAQC (lacking the entire C-terminus, which includes the AQ-rich and the acidic tail). The purified recombinant proteins are shown in Figure 4B.

**Figure 5:**
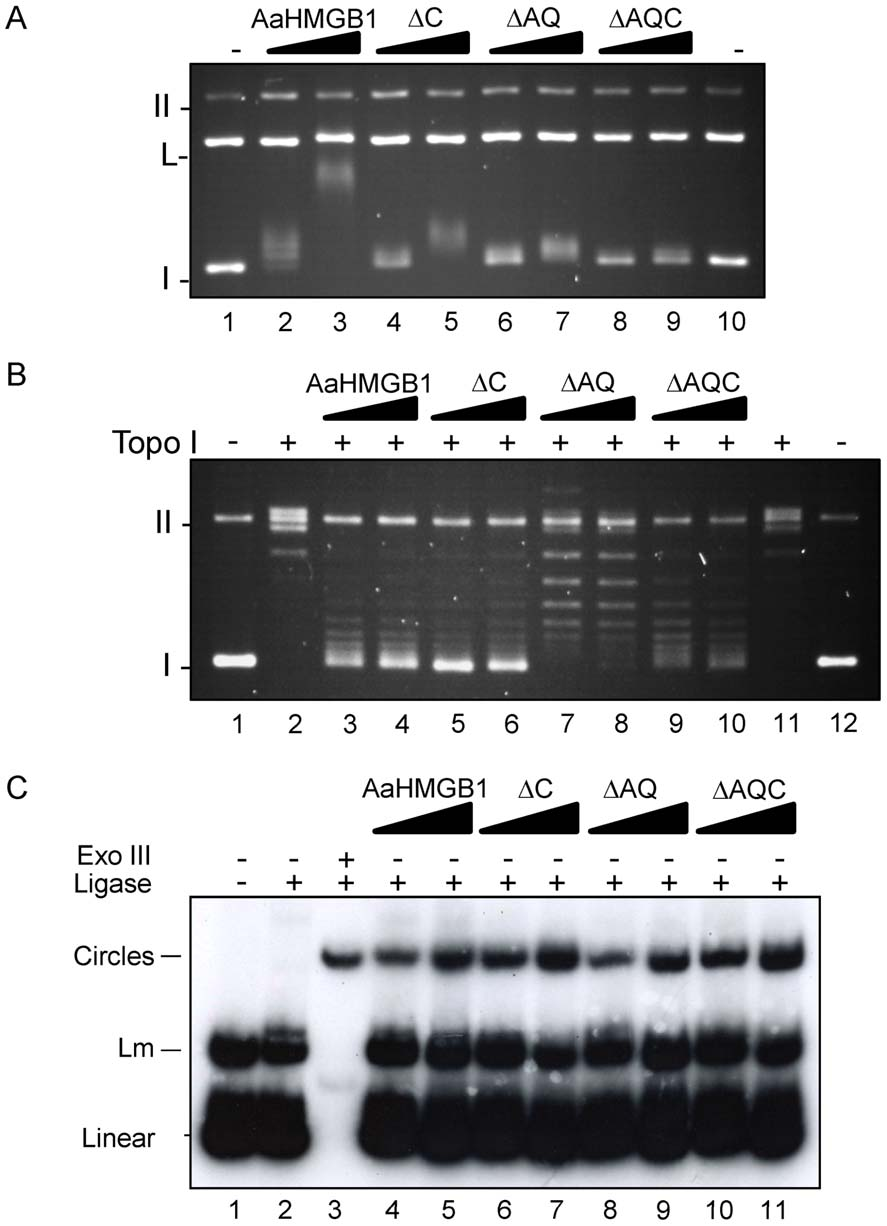
DNA transactions by recombinant AaHMGB1 proteins. (A) Preferential binding of AaHMGB1 protein to supercoiled DNA. An equimolar mixture of supercoiled and linearized plasmid pTZ19R (∼10 nM) was pre-incubated with increasing amounts of AaHMGB1 (0.5–1 µM) and the DNA–protein complexes were resolved on a 1% agarose gel, followed by staining of the gel with ethidium bromide. Form I, supercoiled DNA; L, Linear DNA; Form II, relaxed circular DNA; (B) DNA supercoiling by AaHMGB1 and its truncated forms. Circular relaxed plasmid pTZ19R DNA was incubated in the presence of topoisomerase I (Topo I) and AaHMGB1 recombinant proteins (7–14 µM). Deproteinized DNA topoisomers were resolved on 1% agarose gels, followed by staining of the gel with ethidium bromide. Form I, supercoiled DNA; Form II, relaxed circular DNA. (C) DNA bending by AaHMGB1 and its truncated forms. A ^32^P-labeled 123-bp DNA fragment (∼1 nM) was pre-incubated with recombinant proteins (25–50 nM) followed by ligation with T4 DNA ligase. Exonuclease III was used to verify the identity of DNA circles. The deproteinized DNA ligation products were subjected to electrophoresis on 6% non-denaturing polyacrylamide gels and visualized by autoradiography. Lm: linear multimers. Exo III, exonuclease III. These experiments were repeated three to five times each.

Differently from canonical transcription factors, HMGB1 proteins do not exhibit DNA-sequence specificity. Alternatively, HMGB1 shows a remarkably high affinity for distorted DNA conformations such as supercoiled DNA, four-way-junction DNA and DNA bulges, but it can also actively distort DNA by bending or supercoiling and changing DNA topology [5]. In this paper we used some of these DNA substrates to determine whether AaHMGB1 was active and able to alter DNA conformations. By performing gel retardation assays with complexes prepared by pre-incubation of AaHMGB1 with a mixture of negatively supercoiled (∼45%), linearized (∼45%) and a relaxed closed-circular (∼10%) plasmid pTZ19R, we showed that AaHMGB1, as well as its C-terminal truncation forms exhibited a clear preference binding to supercoiled DNA over linear or relaxed closed-circular DNA (Figure 5A, see the shifted band of supercoiled DNA, lanes 2 and 3). Importantly, sequential removal of the AaHMGB1 acidic tail, the AQ-rich region only and the AQ-rich and acidic tail led to a gradual loss of their affinity to supercoiled DNA (Figure 5, lanes 4–5, 6–7 and 8–9, respectively), with a minimal binding affinity after removal of the entire C-terminal region (lanes 8–9).

HMGB1 proteins can unwind and distort DNA by insertion of negative supercoils (in the presence of topoisomerase I) in topologically closed domain of DNA [32]. Here we analyzed the ability of AaHMGB1 and its truncated forms to induce DNA supercoiling. AaHMGB1 could supercoil DNA in the presence of topoisomerase I (Figure 5B, lanes 3–4). While the removal of the acidic tail only slightly increased AaHMGB1 DNA supercoiling activity (Figure 5B, lanes 5–6), a remarkable loss of activity was observed when the AQ-rich region was deleted (Figure 5B, lanes 7–8). Interestingly, removal of the whole C-terminal region was able to partially restore the supercoiling activity of AaHMGB1 (Figure 5B, lanes 9–10).

A remarkable property of proteins containing HMG-box motifs, including HMGB1, is that these proteins not only bind with high affinity to non-B type DNA conformations, but they also actively distort DNA by bending/looping and unwinding, and changing DNA topology [4], [10], [33]. HMGB1 proteins have been previously shown to bend DNA using the ligase-mediated circularization assay (the DNA ring closure assay) [33]. This assay measures the efficiency with which T4 DNA ligase forms circles from DNA fragments shorter than the persistency length (∼150 bp). In the absence of internal curvature, the stiffness of a short DNA fragment prevents intramolecular alignments so that DNA circles are detected only by a protein that bends DNA. The T4-ligase mediated assay was performed with AaHMGB1 and its C-terminal truncated forms and a 123 bp DNA fragment bearing sticky ends at low concentrations (∼1 nM) to promote intramolecular alignments at the expense of intermolecular associations. The identity of DNA circles was verified by their resistance to Exonuclease III, which digests only linear DNA molecules. As shown in Figure 5C, ligation of the 123 bp-DNA by T4 DNA ligase in the presence of AaHMGB1 or its C-terminal truncated forms (ΔC, ΔAQ and ΔAQC), resulted in the formation of DNA circles. Although in this particular experiment we see a significant lower DNA bending activity of the protein lacking just the AQ-rich region (AaHMGB1-ΔAQ, Figure 5C, lanes 8 and 9; compare lanes 4 and 5, with lanes 8 and 9), densitometry analyses of other five independent DNA bending experiments did not show significant differences. Removal of the whole C-terminus (AaHMGB1-ΔAQC) or just the acidic tail (AaHMGB1-ΔC) did not significantly affect the DNA bending activities of AaHMGB1 proteins (Figure 5C, compare lanes 4 and 5 with lanes 6 and 7, 10 and 11).

### Structural Analysis of Recombinant AaHMGB1 Proteins

**Figure 6:**
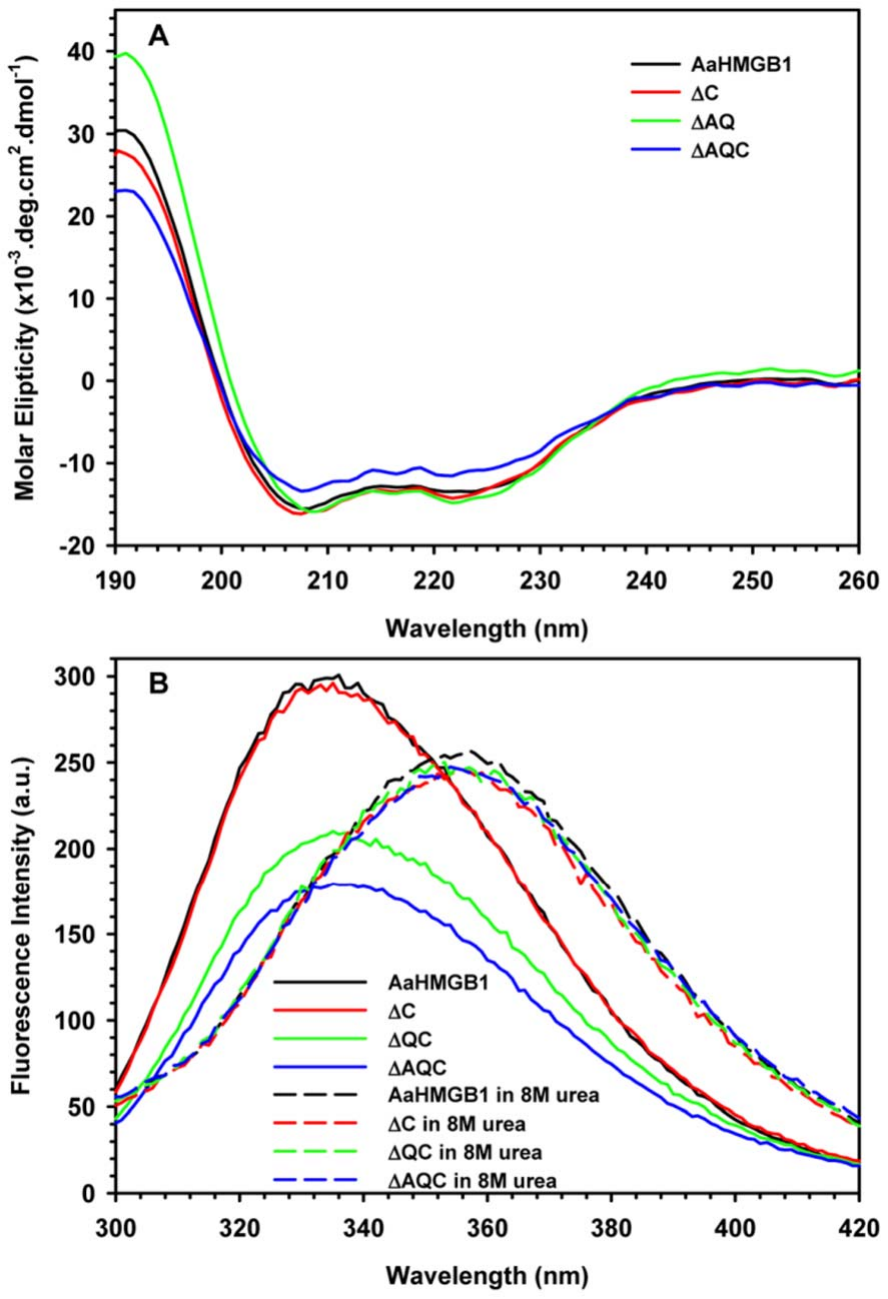
Analysis of secondary and tertiary structures of AaHMGB1 proteins. (A) CD spectra of AaHMGB1 (black line), ΔC (red line), ΔQC (green line) and ΔAQC (blue line) were performed at 25°C. Spectra were averaged from three scans at a 30 nm/min speed recorded from 190 to 260 nm, and the buffer baselines were subtracted from their respective sample spectra. (B) AaHMGB1 (black line), ΔC (red line), ΔQC (green line) and ΔAQC (blue line) were analyzed using fluorescence spectroscopy, either in the absence (native state, solid lines) or presence of 8 M urea (denatured state, dashed lines), in order to evaluate tertiary structure content. The excitation wavelength was fixed at 280 nm and the emission spectrum was recorded from 300 nm to 420 nm. Experiments were performed at 25°C.

To evaluate whether the sequential removal of the C-terminal region of AaHMGB1 protein would compromise its secondary or tertiary structures, conformational changes were monitored by CD and fluorescence spectroscopy. The CD spectra of the proteins displayed negative peaks at 208 and 222 nm, typical of α-helical proteins (Figure 6A). The AaHMGB1 protein lacking its entire C-terminus (AaHMGB1-ΔAQC) displayed a CD spectrum with a slight decrease in the molar ellipticity (Figure 6A, blue line). Alternatively, AaHMGB1 or its other constructs lacking either the short acidic tail or only the AQ-rich region displayed practically an overlapping spectrum (Figure 6A, black, red and green lines), indicating no change in their secondary structure.

The tertiary structure of AaHMGB1 was analyzed by fluorescence spectroscopy of the Tryptophan (Trp) residue (Figure 6B). Tryptophan is an excellent intrinsic fluorescence probe because its fluorescence spectrum shifts to higher wavelengths as it becomes more exposed to solvent [34]. Thus, the three Trp residues present in the AaHMGB1 primary sequence (Trp80, contained within the HMG box A; Trp141 and Trp170, within the HMG box B) were used as intrinsic probes. The Trp fluorescence spectra of all AaHMGB1 proteins showed maximum intensities at a wavelength of about 336 nm (Figure 6B), indicating that these residues were present in a hydrophobic environment, thus suggesting that the protein was assuming a folded conformation. The fluorescence intensity of AaHMGB1 decreased upon sequential removal of its C-terminal region (Figure 6B, compare the black line with the red, green and blue lines), reaching its maximum decrease when the entire C-terminus (AaHMGB1-ΔAQC) was deleted (Figure 6B, blue line). The increase of fluorescence quenching suggested that regions of the C-terminal portion of the protein might be interacting with one of both HMG-box motifs. Alternatively, when incubated in the presence of 8 M urea, the maximum intensity peak of fluorescence spectra of all the proteins shifted to 356 nm, indicating that they were fully denatured in this condition (Figure 6B, dashed lines).

### *In vitro* Phosphorylation Analysis of AaHMGB1

**Figure 7:**
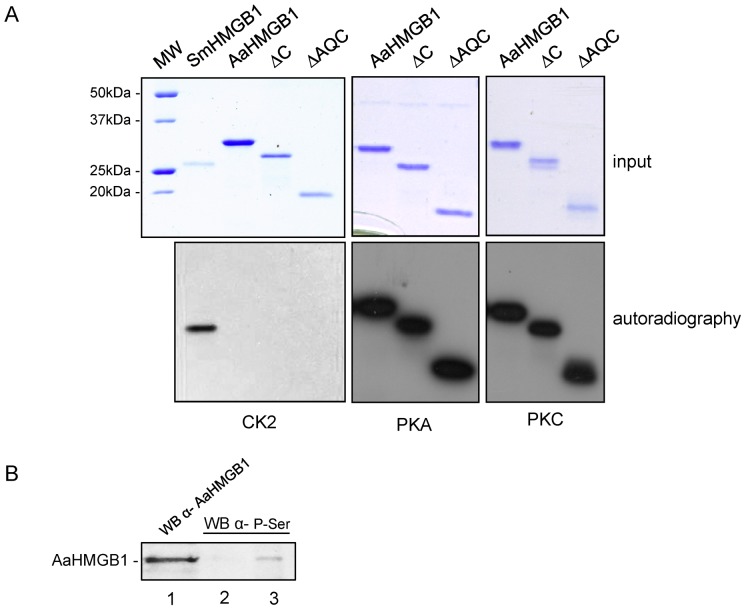
*In vitro* phosphorylation of AaHMGB1. (A) One microgram of AaHMGB1 proteins were subjected to an *in vitro* kinase assay with commercial kinases (CK2, PKA and PKC) and radiolabeled [γ-^32^P] ATP. Phosphorylations were analyzed by 12% SDS-PAGE (top panel) and autoradiography (bottom panel). *Schistosoma mansoni* HMGB1 (SmHMGB1) was used as a positive control for CK2 phosphorylation [37]. (B) Immunoprecipitation of endogenous phosphorylated AaHMGB1. Total protein extract from adult mosquitoes were immune precipitated with pre-immune serum or anti-HMGB1 antibody (lane 3). Western blot analysis was carried out with anti-phospho serine monoclonal antibody (lanes 2 and 3). Endogenous AaHMGB1 (from the protein extract) was reacted against polyclonal anti-AaHMGB1 antibody (lane 1).

HMGB1 from different organisms (mammalian, insect, helminth and plant) have been shown to be phosphorylated by different kinases [14], [35], [36]. We then asked whether AaHMGB1 could also be phosphorylated. Firstly, we subjected the full length amino acid sequence of AaHMGB1 to an *in silico* analysis using the software NetPhosK 1.0 server (http://www.cbs.dtu.dk/services/NetPhosK). The program revealed putative phosphorylation sites with high scores for PKC and PKA, at positions Ser-101, Ser-103, Ser-137, all contained within the HMG-box A and B motifs (data not shown). Two putative CK2 sites were also identified by the program, but with lower scores. We performed *in vitro* phosphorylation reactions (Figure 7A) with recombinant AaHMGB1 proteins. We showed that the three constructs (AaHMGB1, ΔC, ΔAQ) were substrates for PKC and PKA (Figure 7A, lower panels), but not for CK2 (Figure 7A, lower panel). To make sure that the lack of CK2 phosphorylation in AaHMGB1 was not due to a possible inactivity of the CK2 aliquot used in the reactions, we phosphorylated the *Schistosoma mansoni* HMGB1 (SmHMGB1 has been previously shown to be a substrate for CK2) [37]. Figure 7A confirms the CK2 phosphorylation of SmHMGB1, but not of AaHMGB1. In order to prove that phosphorylation takes place in the endogenous protein, we immuneprecipitated AaHMGB1 from the mosquito protein extract, using the anti-AaHMGB1 polyclonal antibody. A Western blot with anti-phospho serine monoclonal antibody was then performed. Figure 7B, shows that the pulled-down AaHMGB1 was indeed phosphorylated at serine residue(s).

### DNA Binding Activities by Phosphorylated AaHMGB1

**Figure 8:**
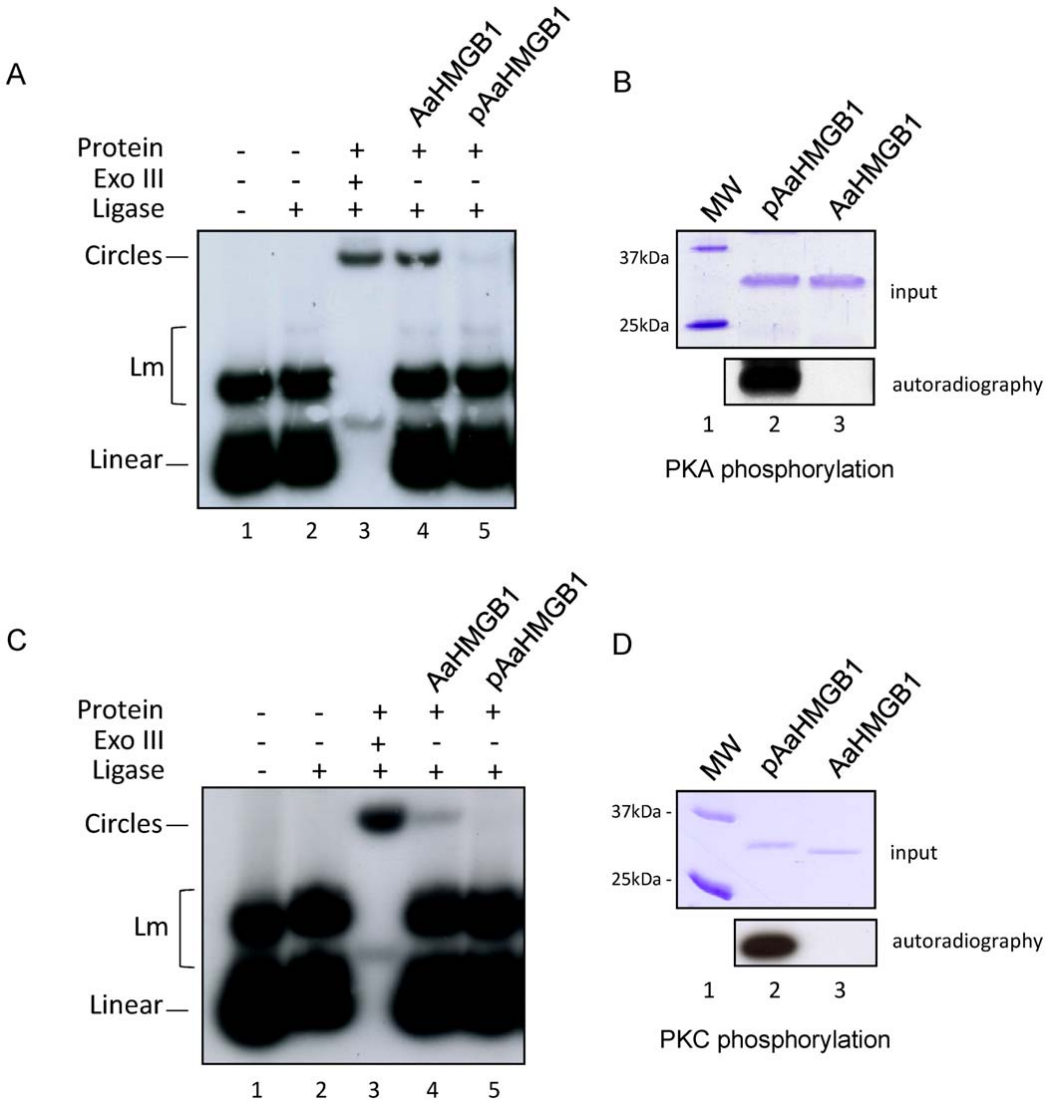
DNA bending assays by posphorylated AaHMGB1. A ^32^P-labelled 123-bp DNA fragment (∼1 nM) was pre-incubated with 50 ng of AaHMGB1 that were phosphorylated by PKA (panels A and B, lanes 5 and 2, respectively) or not (panels A and B, lanes 4 and 3, respectively), or by PKC (panels C and D, lanes 5 and 2, respectively) or not (panels C and D, lanes 4 and 3, respectively), followed by ligation with T4 DNA ligase. Exonuclease III was used to verify the identity of DNA circles. The deproteinized DNA ligation products were subjected to electrophoresis on 6% non-denaturing polyacrylamide gels and visualized by autoradiography. Lm: linear multimers. These experiments were repeated five times.

It has been shown that phosphorylation of HMGB1 proteins modulates not only their DNA binding activities, but also their cellular traffic [35], [36], [37]. In this work we used the well established T4 DNA ligase-mediated circularization assay to determine whether phosphorylation influences the ability of AaHMGB1 to bend DNA. Our data showed that phosphorylation of AaHMGB1 by either PKA (Figure 8A and B) or PKC (Figure 8C and D) almost completely abolished the capacity of AaHMGB1 to bend DNA (Figure 8A and C, compare lanes 4 and 5). Figure 8B and D are controls showing that the AaHMGB1 recombinant proteins used in the reactions were phosphorylated (pAaHMGB1) or not (AaHMGB1). SDS-PAGE shows the integrity of the proteins (Figure 8B and D). We consistently observed an up-shifted migration band in the protein sample that was phosphorylated by PKC (Figure 8D, Coomassie gel, compare lane 2 and 3).

## Discussion

High Mobility Group Box (HMGB) proteins are dynamic chromatin factors endowed with “architectural” capabilities. HMGBs have no sequence specificity and help transcription factors and other nuclear proteins to bind to their cognate sites by bending the DNA molecule. Similarly, HMGBs interact with nucleosomes and fluidize chromatin. Recently, it has been shown that mammalian and yeast cells lacking HMGB1 protein contained a reduced amount of core, linker and variant histones, and a correspondingly reduced number of nucleosomes, possibly because HMGB1 facilitates nucleosome sliding [1]. Because of these (and other) attributes of HMGB proteins, they have emerged as targets for cell survival or cell death.

HMGB proteins have been functionally characterized in only a few insects. In *Chiromonus*, the HMGB protein family is represented by cHMGB1a and cHMGB1b [24], [25]. In *Drosophila*, the HMGB counterparts are HMGD [26], HMGZ [38] and Dsp1 [27]. With the exception of Dsp1, which contains two HMG-box motifs, all other insect HMGB proteins present only one HMG-box [24].

In this study we described structural features and DNA binding properties of a HMGB1 protein found in the mosquito vector, *Aedes aegypti*. Although the *A. aegypti* HMGB1 (AaHMGB1) contains the signature domains of the HMGB protein family (two HMG-boxes and an acidic tail), it also presents unique structural features; for instance, AaHMGB1 contains an alanine-glutamine rich (AQ-rich) domain located just adjacent to its acidic tail.

The acidic C-terminal domain present in vertebrate, insect, plant and unicellular eukaryote HMGB proteins negatively influences various DNA interactions [4], [15], [19], [23], [32], [39]. In this context, the acidic tail of vertebrate HMGB1 down-regulates binding of the two HMGB- boxes to linear DNA and supercoiled DNA, and the ability of the protein to supercoil DNA [5], [10], [32]. The acidic tail of *Drosophila* HMGD modulates binding of the protein to DNA by increasing affinity for bent DNA, decreasing affinity for linear DNA [40] , as well as decreases the efficiency of supercoiling relaxed DNA in the presence of topoisomerase I [40] . For the *Chiromonus* cHMGB1a/b, deletion of the eleven C-terminal acidic residues resulted in an increase of DNA binding affinities of linear and four-way-junction DNA molecules [15]. Overall, our data with the *A. aegypti* HMGB1 corroborate the fact that its acidic tail, although short, displays a negative influence on DNA activities. However, a novel and interesting data was obtained when we assayed the *A. aegypti* protein lacking its unique AQ-rich domain. Removal of the AQ-rich domain from the C-terminus of the protein significantly affected the ability of AaHMGB1 to supercoil DNA in the presence of topoisomerase I, as well as its binding preference for supercoiled DNA (see Figure 5A lanes 6 and 7, and 5B, lanes 7 and 8, respectively ). However, removal of both, the AQ-rich domain and the acidic tail partially restored the supercoiling activity of AaHMGB1, reconfirming the negative effect of the acidic tail moiety in supercoiling DNA. When we tested the effect of AaHMGB1 C-terminal deletions on DNA bending, no changes were observed. In the case of AaHMGB1-ΔC, we observed an important difference when we compared the DNA bending activities of AaHMGB1 and the mammalian HMGB1 [5]. In this regard, our data suggested that the AQ-rich domain of AaHMGB1 does not participate in DNA bending activities.

Analysis of full-length and truncated mammalian and insect HMGB proteins by spectrometric methods has suggested that the acidic tail interacts with the HMG box domains in these proteins [4], [15], [19], [23], [32], [39]. Therefore, intramolecular interactions of the acidic tail and HMG-box domains may modulate DNA interactions of HMGB proteins.

We used CD and fluorescence analysis to evaluate whether the removal of the AQ-rich domain, the acidic tail or the entire C-terminus (the AQ-rich domain and the acidic tail) of AaHMGB1 would compromise protein structure. While the secondary structure of AaHMGB1 was only slightly perturbed when the entire C-terminus was removed, a significant change in the tertiary structure of AaHMGB1 was observed with the AaHMGB1-ΔAQC mutant. These data suggest that, even being short, the acidic tail of AaHMGB1 might be able to interact with its HMG-box (es), and that these interactions might be modulated by the AQ-rich domain.

Phosphorylation of HMGB proteins from different organisms has been shown to play different biological roles, such as DNA binding, cellular traffic and protein folding [5], [35], [36], [37], [40]. Phosphorylation of *Drosophila* HMGD and *Chiromonus* cHMGB1a/b were mapped at multiple serine residues located within their acidic tails [24], [40], [41]. Phosphorylation of HMGD and cHMGB1a/b by CK2 was shown to weaken binding of the proteins to four-way-junction and AT-rich DNA [24], [40]. Differently from the *Drosophila* and *Chiromonus* dipterans, phosphorylation of the *A. aegypti* HMGB1 was accomplished by PKC and PKA, but not by CK2, and likely occurred within its HMG-box motif(s). Importantly, while phosphorylation of HMGD and cHMGB1a/b only slightly weakens its association with DNA, phosphorylation of AaHMGB1 almost completely abolished its ability to bend DNA.

In all organisms studied so far, their HMGB proteins were located in the nucleus. However, it is now well established that HMGB proteins shuttle continually from the nucleus to the cytoplasm, with the equilibrium shifted towards nuclear accumulation [5]. In this respect, we were able to show that AaHMGB1 protein localized mainly in the nuclei of mosquito midgut cells. Interestingly, TEM analysis of mosquito cells containing native AaHMGB1 showed that the bulk of the nuclear proteins were concentrated within heterochromatin regions. Indeed, there are pieces of evidence connecting HMGB proteins to heterochromatin. A yeast two-hybrid screen identified SP100 as an interactor of mammalian HMGB2; SP100 in turn interacts with HP1, the cardinal heterochromatin binding protein, raising the possibility that HMGB2 might be involved in the organization and/or maintenance of heterochromatic regions [42]. In addition, Dsp1 was initially identified in a genetic screen for activities which convert Dorsal into a transcriptional repressor [27]. Subsequent work showed that Dsp1 inhibited activation by the Rel transcription factor Dorsal and NF-kB [28], by a mechanism that depended on the formation of a stable ternary complex containing Dsp1, and the TATA binding protein (TBP) bound to DNA [42]. Thus, Dsp1 preferentially disrupted the DNA binding of TBP complexes containing TFIIA and displaced TFIIA from binding to TBP [43].

Because of the pleiotropic characteristic of HMGB proteins, it is conceivable to imagine AaHMGB1 playing diverse roles in mosquito biology, such as in reproduction, molting and innate immunity. For example, one might envision AaHMGB1 playing an intricately role in vitellogenesis; 20-hydroxyecdysone (20 E) is involved in the control of the vitellogenic period in the mosquito [44], [45], through the action of ecdysone receptors (EcR) [46], which bind to various ecdysteroid response elements to modulate ecdysteroid regulation of target genes [46]. In this regard, mammalian HMGB 1 and 2 functionally interact with steroid receptors to enhance their DNA binding and transcriptional activity [7], [8]. HMGB1/2 facilitate receptor binding to cognate target DNA sites by increasing the flexibility of DNA, thus assembling nucleoprotein complexes involved in regulation of transcription [4], [47].

The discovery of a novel dipteran HMGB protein, typified by AaHMGB1, emphasizes the structural and functional conservation, but also variability, in the HMGB protein family. On the basis of our studies, it is likely that different insect HMGB proteins have specialized functions for assisting the regulation of various DNA-dependent processes.

## Materials and Methods

### Ethics Statement

Animals used for polyclonal antibody production were handled following the guidelines of the institutional care and use committee (Committee for Evaluation of Animal Use for Research from the Federal University of Rio de Janeiro, CAUAP-UFRJ). The protocols were approved by CAUAP-UFRJ under registry IBqM # 030. The procedure was conducted adhering to the institutiońs guidelines for animal husbandry.

### Mosquito Rearing

*Aedes aegypti* (black-eyed Liverpool strain) were maintained in an insectary at the Federal University of Rio de Janeiro, under a 12 h light/dark cycle at 26–30°C and 60–80% relative humidity. Larvae were reared in 20×30×5 cm pans with filtered water and fed with commercial dog food (Pedigree Junior, Masterfoods Brasil Alimentos Ltda). Adult mosquitoes were maintained in a cage and given a solution of 10% sucrose *ad libitum*.

### Bioinformatics

Arthropod sequences were extracted from NCBI non-redundant (*nr*) database using a Perl script developed by Dr. Rafael Dias Mesquita (UFRJ). This program: 1) Search all branches bellow a taxonomic point using NCBI taxonomic index (ftp://ftp.ncbi.nih.gov/pub/taxonomy/taxdump.tar.gz) and gi-taxonid relation database (ftp://ftp.ncbi.nih.gov/pub/taxonomy/gi_taxid_prot.dmp.gz); and 2) select and extract from *nr* all sequences related to any branch previously listed. Arthropod taxonid 6656 was used to create the initial dataset.

For HMGB identification, we used the FAT software [48], which integrates HMMER (http://hmmer.janelia.org/) and BLAST+ tools [49] to filter the initial dataset and perform automatic annotation. The filter step used the HMG-box conserved domain (Pfam code PF00505) to identify and extract only proteins containing such a domain on the arthropods dataset. The annotation step compared the filtered proteins for similarity with proteins and conserved domains databases using respectively a) BLAST with *nr* and *Swiss-prot uniprot* databases and b) HMMSCAN software with *Pfam* database [50]. All results were manually inspected. Seven mammalian HMGB1 proteins (*Mus musculus, Homo sapiens, Equus caballus, Canis lupus familiaris, Callithrix jacchus, Bos taurus* and *Sus scrofa*), were selected from Swiss-Prot database. These sequences were often found as top hits in the identified arthropod HMGB1 BLAST results when *Swiss-prot* was used as comparison database.

For phylogenetic analysis, HMGB1 protein sequences were aligned using CLUSTALW [51]. PHYLIP package [52] was used for phylogeny calculations. Protein distance matrix and dendograms were calculated using PROTDIST and NEIGHBOR, respectively. Bootstrap analysis used a 10,000 replicates dataset created with SEQBOOT and analyzed as above, before support calculation using CONSENSE. All programs were used with standard parameters. Trees and dendograms were created using FIGTREE and edited with GIMP. The figure of the multiple alignments was colored with BIOEDIT.

### RNA Isolation and Real Time PCR

Total RNA was isolated from all developmental stages (egg, larvae 1–4, pupae, male and female adult mosquitoes that were sugar fed). Total RNA was extracted using TRIzol reagent (Invitrogen). Genomic DNA contamination was removed by treatment with RNase-free-DNase I (Fermentas) prior to cDNA synthesis. Reverse transcription was carried out using RevertAid™ H Minus M-MuLV Reverse Transcriptase kit (Fermentas) in a 20 µL reaction mix containing random primers (Invitrogen) and 1 µg of total RNA. Quantitative PCR (qPCR) was carried out in an ABI PRISM 7500 Sequence Detection System (Applied Biosystem). Amplifications were performed in 15 µL reaction containing 1× Power SYBR Green PCR Master Mix (Applied Biosystem) and 0.3 µM of each primer. Primers were as follows: AaHMGB1 sense, –5′ ATCACTGTCGGCGTTCTTCT 3′; AaHMGB1 anti-sense, –5′ CCCTGTTCGTTTTGCATTT 3′. Quantifications were are relative to the expression of the housekeeping gene rp49 (*A. aegypti* ribossomal 49 gene) [53]. All experiments were performed using three biological replicates and three experimental replicates. The relative quantification of AaHMGB1 was determined using the comparative Ct method, also known as the ΔΔCt method [54]. Cycling conditions were: RT step at 50°C for 2 min, initial denaturation at 95°C for 10 min, and 40 cycles at 95°C for 15 s, at 60°C for 1 min. Data analysis was carried out using Excel software (Microsoft, USA) and the statistical analysis were done by one-way ANOVA and the *posteriori* Tukey’s test using the GraphPad Prism5 for Windows (GraphPad Software, USA). Differences of p<0.05 were considered as significant.

### Plasmids

The cDNAs enconding all proteins used in this work were RT-PCR amplified using the sense primer 5′***GGATCC***ACGACGAACGGAACAAGGTTA 3′ (*Bam*HI restriction site is in italic and bold); the anti-sense primers were as follows: for AaHMGB1∶5′***AAGCTT***TTACTCGTTATCATCCTCG 3′; for AaHMGB1ΔC: 5′***AAGCTT***GGCGTGGTGTGCCG 3′: for AaHMGB1-ΔAQC: 5′ ***AAGCTT*** GTACTCGGTCATTTCCTG 3′ (*Hind*III restriction sites are in italic and bold). RT-PCR products were cloned into pGEM T-Easy vector (Promega) and sequenced on both strands. In order to generate recombinant his-tagged proteins, plasmids were digested with the appropriate enzymes (Promega) and cloned into the pQE-80L expression vector (Qiagen), according to the manufacturer’s instructions. For the construction of AaHMGB1-ΔAQ, pQE-80L/AaHMGB1 was transformed into XL1blue strain, the plasmid was purified and used as a template in PCR reactions (sense primer 5′ TTTGCATTTTAGCTTGTACTCGG 3′ and anti-sense primer 5′ GACGATGACGATGATGACGTCGAC 3′) with Elongase enzyme (Invitrogen). The PCR product was gel purified, treated with *Dpn*I, ligated and retransformed into BL21 strain. The identity of the clone was confirmed by sequencing.

### Expression of Recombinant Proteins and Production of Polyclonal Antibody

Recombinant proteins (AaHMGB1, aa 35–244; AaHMGB1-ΔC, aa 35–231; AaHMGB1-ΔAQ, aa 171 to 231 were deleted; and AaHMGB1-ΔAQC, aa 35–186) were expressed fused with 6 histidines (6×-His-tag) at their N-termini as previously described [21] **.** Protein concentration was determined by the Bio-Rad Protein Assay (Bio-Rad). The purity of AaHMGB1 recombinant proteins was checked by 12% SDS-PAGE, followed by Coomassie Blue R-250 staining (Figure 4B). Polyclonal mouse serum was produced against preparations of recombinant AaHMGB1. Mice were inoculated with 50 µg of protein mixed with complete Freunds adjuvant (Sigma) and boosted four times with 50 µg of protein mixed with incomplete Freunds adjuvant (Sigma). Pre-immune serum was collected before the first immunization.

### Phosphorylation Assays

Recombinant AaHMGB1 proteins (1 µg) were phosphorylated by commercial rat protein kinase CK2 (Promega), human protein kinase A (PKA) (Millipore) or rat protein kinase C (PKC) (Promega). Reactions were carried out in CK2 buffer (25 mM Tris-HCl, pH 7.4, 200 mM NaCl, 10 mM MgCl_2_, and 0.1 mM ATP), at 37°C, PKA buffer (100 mM HEPES, pH 7.0, KCl 200 mM, 20 mM MgCl_2_, 0.1 mM ATP), at 30°C or PKC buffer (30 mM Tris-HCl, pH 7.6, 2 mM dithiothreitol, 6 mM Mg(CH_3_COO)_2_, 0.4 mM CaCl_2_, 0.6 µg 1,2-Diacyl-*sn*-glycero-3-phospho-L-serine, 0.12 mM ATP), at 30°C. Reactions were carried out for one hour in the presence of 0.5 µCi γ^32^P [ATP] (PerkimElmer). The reaction was stopped by adding SDS-PAGE sample buffer (50 mM Tris-HCl pH 6.8, 2% SDS, 0.1% bromophenol blue, 10% glycerol and 100 mM dithiothreitol). For the unphosphorylated control reactions, proteins were incubated in phosphorylation reactions lacking the protein kinase. The phosphorylation status of the proteins was examined by autoradiography and protein input controls were examined by Coomassie Blue R-250 staining.

### Immunopreciptation and Western Blot

*A. aegypti* total protein extract were carried out by homogenizing adult mosquitoes in TBS 1×, containing a protease inhibitor cocktail (SIGMA). Proteins were recovered from the supernatant by centrifugation at 14.000×g, 15 min. at 4°C. Protein concentration was determined by the Bio-Rad Protein Assay (Bio-Rad). The AaHMGB1 was immunoprecipitated (following the manufacturer’s instructions; Protein-A/G Plus-Agarose, Santa Cruz Biotechnology), from 500 µg of total protein extract using the anti-AaHMGB1 antibody. Controls for the immunoprecipitation using the pre-immune serum or only the Protein A/G Agarose (not shown) were carried out. Western blots were carried out using a polyclonal antibody against AaHMGB1 or a monoclonal antibody against phosphoserine (Millipore).

### DNA Supercoiling Assay

DNA supercoiling assays were carried out as previously described [21]. Briefly, CsCl-purified supercoiled plasmid pTZ19R was relaxed at a DNA concentration ∼170 µg/mL in Topoisomerase I (Topo I) relaxation buffer (50 mM NaCl, 50 mM Tris–HCl, pH 7.5, 1 mM EDTA, 20% glycerol and 1 mM dithiothreitol) in the presence of Topo I (2 units/µg DNA; Promega) at 37°C for 90 min. The relaxed DNA (0.5 µg DNA) was then diluted to final 140 mM NaCl, then the same amount of the Topo I was added, followed by the addition of recombinant AaHMGB1 proteins (7–14 µM). The 20 µL reactions were allowed to proceed at 37°C for 60 min after which they were terminated by addition of SDS and NaCl to final 1% and 1 M, respectively. DNA was deproteinized by chloroform/isoamyl alcohol (24∶1) extraction in the presence of 0.02% linear polyacrylamide (LPA, Sigma). Deproteinized DNA was then precipitated with 2.5 volume of ethanol, washed with 70% ethanol, air-dried and finally dissolved in TE buffer. The occourance of DNA topoisomers was analyzed by electrophoresis in 1% agarose gels in 1× TBE buffer at 3 V/cm for 17 h. The gels were stained with 0.5 µg/ml ethidium bromide, distained in water and photographed through a red filter in an UV-transilluminator (Mini-Bis Pro, Bio Imaging Systems).

### T4 DNA Ligase-mediated Circularization Assay

The circularization assay (or bending assay) was carried out as previously described [21]. Briefly, a ^32^P-labeled 123-bp DNA fragment (∼1 nM) with cohesive BamHI ends were pre-incubated on ice for 20 min with appropriate amounts of recombinant proteins (25–50 nM) or total protein extracts from adult mosquitos (4 µg) in 1× T4 DNA ligase buffer (30 mM Tris–HCl, pH 7.8, 10 mM MgCl_2_, 10 mM dithiothreitol, and 0.5 mM ATP; Promega) in a final volume of 20 µL. The DNA was then ligated with T4 DNA ligase (0.6 unit/reaction; Promega) at 30°C for 30 min, and the ligation reactions were terminated by incubation of samples at 65°C for 15 min. Some of the ligation mixtures were digested after termination of ligations with ∼25 units of Exonuclease III (Promega) at 37°C for 30 min. Before electrophoresis, all DNA samples were deproteinized as described in the DNA supercoiling assay. The protein-free DNAs were loaded on pre-run 6% polyacrylamide gels in 0.5× TBE buffer, and finally resolved at 200 V for 3 h at 4°C. After electrophoresis, the gels were vacuum-dried and visualized by autoradiography.

### Gel Retardation Assay

Gel retardation experiments were carried out as using an equimolar mixture of highly-purified negatively supercoiled plasmid pTZ19R and the HindIII-linearized plasmid, as previously described [55]. Briefly, 0.5 µg of plasmid DNAs was mixed with increasing amounts (0.5–1 µM) of recombinant proteins in buffer A (0.14 M NaCl, 20 mM Tris/HCl, pH 7.5, 0.2 mM EDTA, 5 mM DTT) in a final volume of 20 µL and pre-incubated on ice for 30 min. The DNA–protein complexes were resolved by electrophoresis on 1% agarose gels in 0.5× TBE buffer at 3 V/cm for 17 h at 4°C. The gels were stained with 0.5 µg/mL ethidium bromide, distained in water and photographed through a red filter in an UV-transilluminator (Mini-Bis Pro, Bio Imaging Systems).

### Circular Dichroism and Fluorescence Spectroscopy

The circular dichroism (CD) experiments were conducted in a Chirascan Circular Dichroism Spectropolarimeter (Applied Photophysics, UK) at 25°C using a quartz cuvette with a 0.01 cm path length. Spectra from three scans at a 30 nm/min speed and scanned from 190 to 260 nm were averaged, and the buffer baselines were subtracted from their respective sample spectra. Measurements of the molar ellipticity were carried out in 10 mM Tris.HCl pH 7.5, 50 mM NaCl, 0.5 mM DTT, 0.1 mM EDTA, and 5% glycerol. The final protein concentration of each sample used in the measurements was quantitated by Bradford Assay kit (Sigma) and shown to be at 25 µM.

The fluorescence spectroscopy measurements were performed in a Varian Cary Eclipse spectrofluorometer (Australia). The excitation wavelength was fixed at 280 nm, and the emission spectrum was recorded from 300 to 420 nm, using slits of 5/5 nm in the excitation and emission paths, respectively. A 1-cm path length quartz cuvette was used. All the experiments were performed at 25°C in a buffer containing 10 mM Tris.HCl pH 7.5, 50 mM NaCl, 0.5 mM DTT, 0.1 mM EDTA, and 5% Glycerol, in the absence or presence of 8 M urea. The final protein concentration of each sample used in the measurements was quantitated by Bradford Assay kit (Sigma) and shown to be at 2 µM.

### Immune-histochemistry

Mosquitós midguts were dissected into 1× PBS 24 h after sugar feeding and fixed in 4% paraformaldehyde for 1 hour. The midguts were blocked in PBT solution (1× PBS, 1% BSA, 0.1% Triton X-100) for 3 hours at room temperature followed by 16 hours incubation with primary antibodies at 4°C with gentle rocking. The midguts were subsequently washed 3× with PBT for 20 min at room temperature and incubated with an Alexa Fluor 488 conjugated anti-mouse (Invitrogen) for 1 hour at room temperature. The sections were mounted in prolong® Gold antifade reagent with DAPI (Invitrogen). The experiments were repeated three times, and representative images were taken by Zeiss Axio Observer.Z1 invert microscope equipped with 100× objective lens and an AxioCam MRm camera, in the ApoTome mode. Negative controls were obtained using only the secondary antibody (not shown) or the pre-immune serum (Figure S2).

### Cell Culture

*Aedes albopictus* C6/36 embryonic cell line (CRL-1660™, ATCC) were seeded in 25 cm^2^ plastic flasks and grown at 28°C in Leibovitz medium (L-15; Gibco). 29.5 g/L L-15, 10% of tryptose phosphate broth (Sigma), 0.75% sodium bicarbonate (Sigma), 0.2% of l-glutamine (Sigma), 5% MEMnon-essential amino acid solution (Sigma), Milli-Q water.

### Transmission Electron Microscopy

The samples were fixed overnight at room temperature in 0.5% glutaraldehyde, 4% formaldehyde in 0.1 M cacodylate buffer. Afterwards, the cells were washed in PBS and post-fixed for 15 min in 0.1% OsO_4_ in 0.1 M cacodylate buffer containing 5 mM CaCl_2_ and 0.8% of potassium ferricyanide. The cells were dehydrated in ethanol and embedded in Epon. Ultra-thin sections were harvested on 300 mesh copper grids, stained with 5% uranyl acetate and 1% lead citrate and then observed with a JEOL 1210 transmission electron microscope.

### Immunolabelling

The cells were fixed overnight in 0.5% glutaraldehyde, 4% formaldehyde in 0.1 M cacodylate buffer, pH 7.2, dehydrated in ethanol and embedded in Epon. For immunolabelling, thin sections were incubated in 1% H_2_O_2_. Afterwards, the samples were quenched in 50 mM NH_4_Cl for 30 min and incubated with primary antibody anti-HMGB1. After several washes in PBS-1% albumin, sections were incubated with 15 nm gold-conjugated anti-mouse IgG (BB International, UK). The samples were washed and observed with a JEOL 1210 electron microscope. Controls included samples incubated with the pre-immune serum only or without the primary antibody.

## Supporting Information

Figure S1 **Alignment of deduced amino acid sequences of HMGB1 proteins.** HsHMGB1 (*homo sapiens*, accession number EAX08458), Dsp1 (*Drosophila melanogaster,* accession number NP_001138203), AaHMGB1 (*Aedes aegypti,* accession number XP_001655323). The HMG box A (aa 39 to 114) and HMG box B (aa 127 to 200) are highly conserved (identical and conserved amino acids are shaded in black and grey, respectively). The black line depicts the region with putative nuclear localization signals (NLS). The red line depicts the unique AQ-rich domain.
(TIF)

Figure S2 **Control for the immune-histochemistry.** Immunostaining with the pre-immune serum in the midguts of adult sugar-fed mosquitoes. Nuclei were stained with DAPI. Scale bar: 20 µm.
(TIF)

Table S1 **Description of Orders containing the AQ-rich C-terminal domain.** HMGB1-containing organisms were separated by their Orders and analyzed for the presence of an AQ-rich domain in their C-terminus. Note that among the insects, the presence of an AQ-rich domain in the C-terminus of HMGB1 proteins is a peculiarity of the dipterans. Such a domain does not exist in mammals either. The different Orders are represented by the different colors. Asterisks indicate infraorder or suborder, when Orders were not found in the NCBI taxonomy database.
(TIF)

## References

[1] Celona B, Weiner A, Di Felice F, Mancuso FM, Cesarini E (2011). Substantial histone reduction modulates genomewide nucleosomal occupancy and global transcriptional output.. PLoS Biol.

[2] Li B, Carey M, Workman JL (2007). The role of chromatin during transcription.. Cell.

[3] Groth A, Rocha W, Verreault A, Almouzni G (2007). Chromatin challenges during DNA replication and repair.. Cell.

[4] Thomas JO, Travers AA (2001). HMG1 and 2, and related ‘architectural’ DNA-binding proteins.. Trends Biochem Sci.

[5] Stros M (2010). HMGB proteins: interactions with DNA and chromatin.. Biochim Biophys Acta.

[6] Bianchi ME, Agresti A (2005). HMG proteins: dynamic players in gene regulation and differentiation.. Curr Opin Genet Dev.

[7] Boonyaratanakornkit V, Melvin V, Prendergast P, Altmann M, Ronfani L (1998). High-mobility group chromatin proteins 1 and 2 functionally interact with steroid hormone receptors to enhance their DNA binding in vitro and transcriptional activity in mammalian cells.. Mol Cell Biol.

[8] Roemer SC, Adelman J, Churchill ME, Edwards DP (2008). Mechanism of high-mobility group protein B enhancement of progesterone receptor sequence-specific DNA binding.. Nucleic Acids Res.

[9] Martin D, Daulny A, Decoville M, Locker D (2003). Mutagenesis analysis of the interaction between the dorsal rel homology domain and HMG boxes of DSP1 protein.. J Biochem.

[10] Stros M, Muselikova-Polanska E, Pospisilova S, Strauss F (2004). High-affinity binding of tumor-suppressor protein p53 and HMGB1 to hemicatenated DNA loops.. Biochemistry.

[11] Lotze MT, Tracey KJ (2005). High-mobility group box 1 protein (HMGB1): nuclear weapon in the immune arsenal.. Nat Rev Immunol.

[12] Bianchi ME, Manfredi AA (2007). High-mobility group box 1 (HMGB1) protein at the crossroads between innate and adaptive immunity.. Immunol Rev.

[13] Bonaldi T, Talamo F, Scaffidi P, Ferrera D, Porto A (2003). Monocytic cells hyperacetylate chromatin protein HMGB1 to redirect it towards secretion.. EMBO J.

[14] Youn JH, Shin JS (2006). Nucleocytoplasmic shuttling of HMGB1 is regulated by phosphorylation that redirects it toward secretion.. J Immunol.

[15] Wisniewski JR, Schulze E (1994). High affinity interaction of dipteran high mobility group (HMG) proteins 1 with DNA is modulated by COOH-terminal regions flanking the HMG box domain.. J Biol Chem.

[16] Stott K, Watson M, Howe FS, Grossmann JG, Thomas JO (2010). Tail-mediated collapse of HMGB1 is dynamic and occurs via differential binding of the acidic tail to the A and B domains.. J Mol Biol.

[17] Pasheva E, Sarov M, Bidjekov K, Ugrinova I, Sarg B (2004). In vitro acetylation of HMGB-1 and -2 proteins by CBP: the role of the acidic tail.. Biochemistry.

[18] Briquet S, Boschet C, Gissot M, Tissandie E, Sevilla E (2006). High-mobility-group box nuclear factors of Plasmodium falciparum.. Eukaryot Cell.

[19] Abhyankar MM, Hochreiter AE, Hershey J, Evans C, Zhang Y (2008). Characterization of an Entamoeba histolytica high-mobility-group box protein induced during intestinal infection.. Eukaryot Cell.

[20] Kiilerich B, Stemmer C, Merkle T, Launholt D, Gorr G (2008). Chromosomal high mobility group (HMG) proteins of the HMGB-type occurring in the moss Physcomitrella patens.. Gene.

[21] de Oliveira FM, de Abreu da Silva IC, Rumjanek FD, Dias-Neto E, Guimaraes PE (2006). Cloning the genes and DNA binding properties of High Mobility Group B1 (HMGB1) proteins from the human blood flukes Schistosoma mansoni and Schistosoma japonicum.. Gene.

[22] Thomsen MS, Franssen L, Launholt D, Fojan P, Grasser KD (2004). Interactions of the basic N-terminal and the acidic C-terminal domains of the maize chromosomal HMGB1 protein.. Biochemistry.

[23] Chen YH, Jia XT, Huang XD, Zhang S, Li M (2011). Two Litopenaeus vannamei HMGB proteins interact with transcription factors LvSTAT and LvDorsal to activate the promoter of white spot syndrome virus immediate-early gene ie1.. Mol Immunol.

[24] Aleporou-Marinou V, Marinou H, Patargias T (2003). A mini review of the high mobility group proteins of insects.. Biochem Genet.

[25] Wisniewski JR, Schulze E (1992). Insect proteins homologous to mammalian high mobility group protein 1. Characterization and DNA-binding properties.. J Biol Chem.

[26] Wagner CR, Hamana K, Elgin SC (1992). A high-mobility-group protein and its cDNAs from Drosophila melanogaster.. Mol Cell Biol.

[27] Lehming N, Thanos D, Brickman JM, Ma J, Maniatis T (1994). An HMG-like protein that can switch a transcriptional activator to a repressor.. Nature.

[28] Mosrin-Huaman C, Canaple L, Locker D, Decoville M (1998). DSP1 gene of Drosophila melanogaster encodes an HMG-domain protein that plays multiple roles in development.. Dev Genet.

[29] Salvaing J, Decoville M, Mouchel-Vielh E, Bussiere M, Daulny A (2006). Corto and DSP1 interact and bind to a maintenance element of the Scr Hox gene: understanding the role of Enhancers of trithorax and Polycomb.. BMC Biol.

[30] Lamiable O, Rabhi M, Peronnet F, Locker D, Decoville M (2010). Rm62, a DEAD-box RNA helicase, complexes with DSP1 in Drosophila embryos.. Genesis.

[31] Muller S, Scaffidi P, Degryse B, Bonaldi T, Ronfani L (2001). New EMBO members’ review: the double life of HMGB1 chromatin protein: architectural factor and extracellular signal.. EMBO J.

[32] Stros M, Stokrova J, Thomas JO (1994). DNA looping by the HMG-box domains of HMG1 and modulation of DNA binding by the acidic C-terminal domain.. Nucleic Acids Res.

[33] Stros M (1998). DNA bending by the chromosomal protein HMG1 and its high mobility group box domains. Effect of flanking sequences.. J Biol Chem.

[34] Ward LD (1985). Measurement of ligand binding to proteins by fluorescence spectroscopy.. Methods Enzymol.

[35] Zhang X, Wheeler D, Tang Y, Guo L, Shapiro RA (2008). Calcium/calmodulin-dependent protein kinase (CaMK) IV mediates nucleocytoplasmic shuttling and release of HMGB1 during lipopolysaccharide stimulation of macrophages.. J Immunol.

[36] Oh YJ, Youn JH, Ji Y, Lee SE, Lim KJ (2009). HMGB1 is phosphorylated by classical protein kinase C and is secreted by a calcium-dependent mechanism.. J Immunol.

[37] de Abreu da Silva IC, Carneiro VC, Maciel Rde M, da Costa RF, Furtado DR (2011). CK2 phosphorylation of Schistosoma mansoni HMGB1 protein regulates its cellular traffic and secretion but not its DNA transactions.. PLoS One.

[38] Ner SS, Churchill ME, Searles MA, Travers AA (1993). dHMG-Z, a second HMG-1-related protein in Drosophila melanogaster.. Nucleic Acids Res.

[39] Lee KB, Thomas JO (2000). The effect of the acidic tail on the DNA-binding properties of the HMG1,2 class of proteins: insights from tail switching and tail removal.. J Mol Biol.

[40] Payet D, Travers A (1997). The acidic tail of the high mobility group protein HMG-D modulates the structural selectivity of DNA binding.. J Mol Biol.

[41] Wisniewski JR, Schulze E, Sapetto B (1994). DNA binding and nuclear translocation of insect high-mobility-group- protein-1 (HMG1) proteins are inhibited by phosphorylation.. Eur J Biochem.

[42] Paull TT, Haykinson MJ, Johnson RC (1993). The nonspecific DNA-binding and -bending proteins HMG1 and HMG2 promote the assembly of complex nucleoprotein structures.. Genes Dev.

[43] Lehming N, Le Saux A, Schuller J, Ptashne M (1998). Chromatin components as part of a putative transcriptional repressing complex.. Proc Natl Acad Sci U S A.

[44] Kirov NC, Lieberman PM, Rushlow C (1996). The transcriptional corepressor DSP1 inhibits activated transcription by disrupting TFIIA-TBP complex formation.. EMBO J.

[45] Raikhel AS, Kokoza VA, Zhu J, Martin D, Wang SF (2002). Molecular biology of mosquito vitellogenesis: from basic studies to genetic engineering of antipathogen immunity.. Insect Biochem Mol Biol.

[46] Hagedorn HH (1989). Physiological roles of hemolymph Ecdysteroid in the adult insect..

[47] Wang SF, Miura K, Miksicek RJ, Segraves WA, Raikhel AS (1998). DNA binding and transactivation characteristics of the mosquito ecdysone receptor-Ultraspiracle complex.. J Biol Chem.

[48] Seabra-Junior ES, Souza EM, Mesquita RD, IFRJ (2011). FAT - Functional Analysis Tool..

[49] Camacho C, Coulouris G, Avagyan V, Ma N, Papadopoulos J (2009). BLAST+: architecture and applications.. BMC Bioinformatics.

[50] Finn RD, Mistry J, Tate J, Coggill P, Heger A (2010). The Pfam protein families database.. Nucleic Acids Res.

[51] Thompson JD, Gibson TJ, Higgins DG (2002). Multiple sequence alignment using ClustalW and ClustalX.. Curr Protoc Bioinformatics Chapter 2: Unit 2 3.

[52] Retief JD (2000). Phylogenetic analysis using PHYLIP.. Methods Mol Biol.

[53] Gentile C, Lima JB, Peixoto AA (2005). Isolation of a fragment homologous to the rp49 constitutive gene of Drosophila in the Neotropical malaria vector Anopheles aquasalis (Diptera: Culicidae).. Mem Inst Oswaldo Cruz.

[54] Pfaffl MW, Lange IG, Daxenberger A, Meyer HH (2001). Tissue-specific expression pattern of estrogen receptors (ER): quantification of ER alpha and ER beta mRNA with real-time RT-PCR.. APMIS.

[55] Stros M, Reich J (1998). Formation of large nucleoprotein complexes upon binding of the high-mobility-group (HMG) box B-domain of HMG1 protein to supercoiled DNA.. Eur J Biochem.

