# Supplementary figures and images for: The Dengue Vector Aedes aegypti Contains a Functional High Mobility Group Box 1 (HMGB1) Protein with a Unique Regulatory C-Terminus
Source: PLoS One. 2012 Jul 3;7(7):e40192. doi: 10.1371/journal.pone.0040192 (PMC3388995; doi:10.1371/journal.pone.0040192)

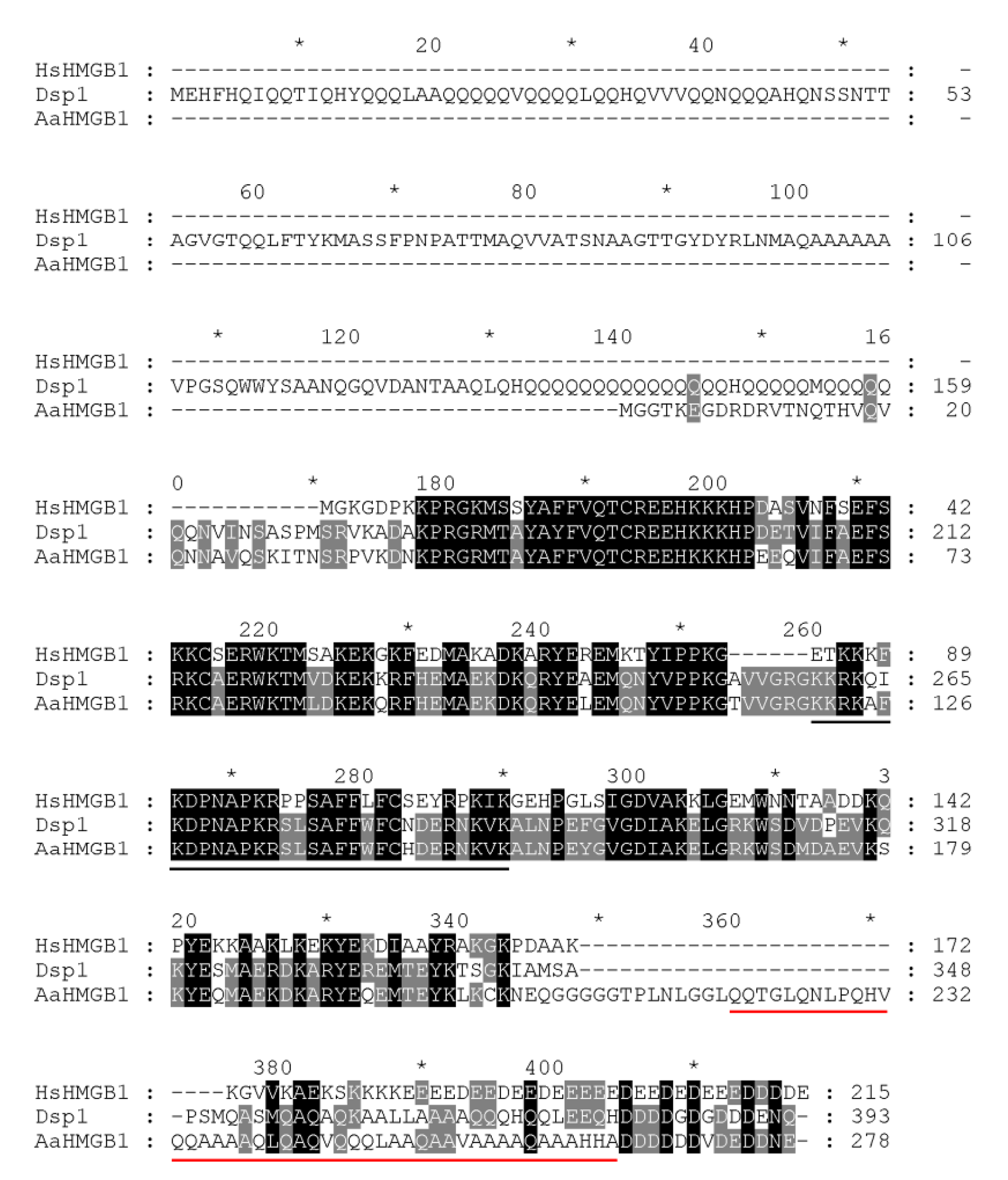

Supplement: Figure S1 — Alignment of deduced amino acid sequences of HMGB1 proteins. HsHMGB1 (homo sapiens, accession number EAX08458), Dsp1 (Drosophila melanogaster, accession number NP_001138203), AaHMGB1 (Aedes aegypti, accession number XP_001655323). The HMG box A (aa 39 to 114) and HMG box B (aa 127 to 200) are highly conserved (identical and conserved amino acids are shaded in black and grey, respectively). The black line depicts the region with putative nuclear localization signals (NLS). The red line depicts the unique AQ-rich domain. (TIF) [file pone.0040192.s001.tif]

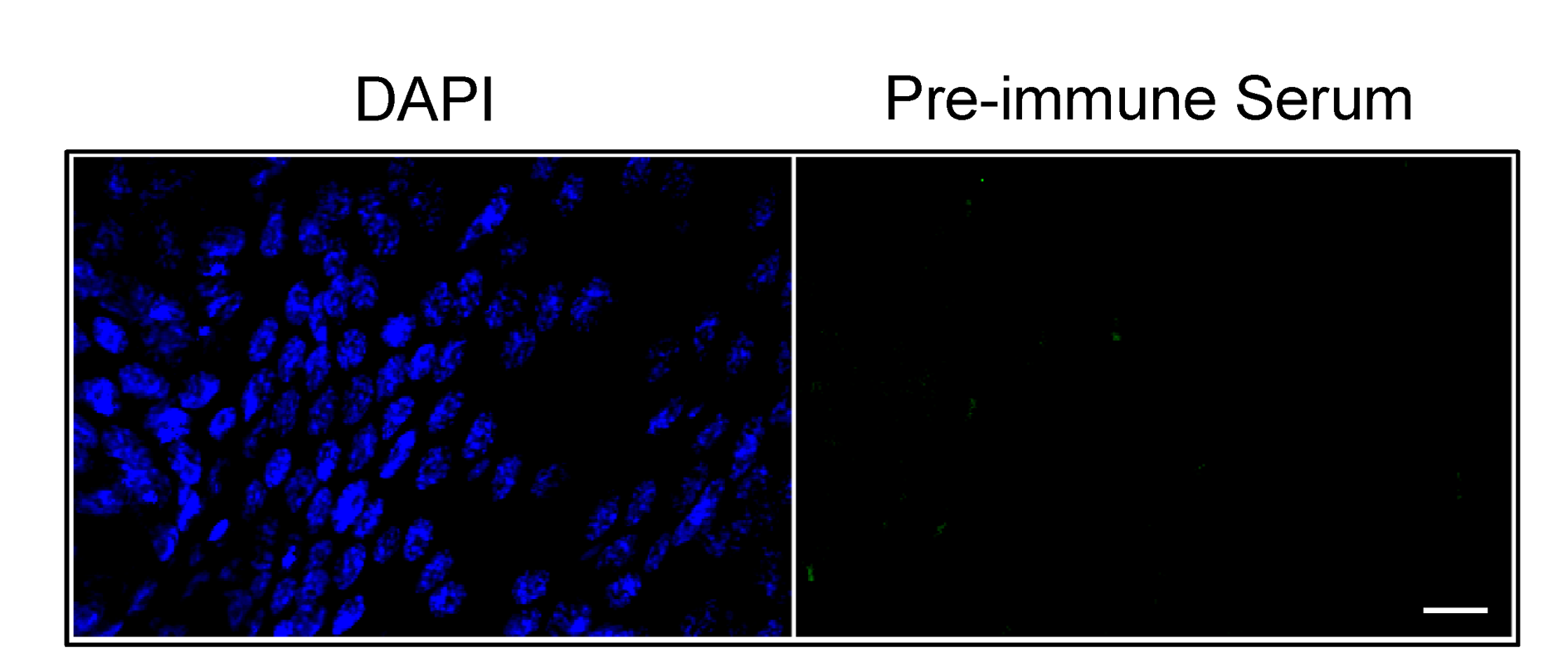

Supplement: Figure S2 — Control for the immune-histochemistry. Immunostaining with the pre-immune serum in the midguts of adult sugar-fed mosquitoes. Nuclei were stained with DAPI. Scale bar: 20 µm. (TIF) [file pone.0040192.s002.tif]

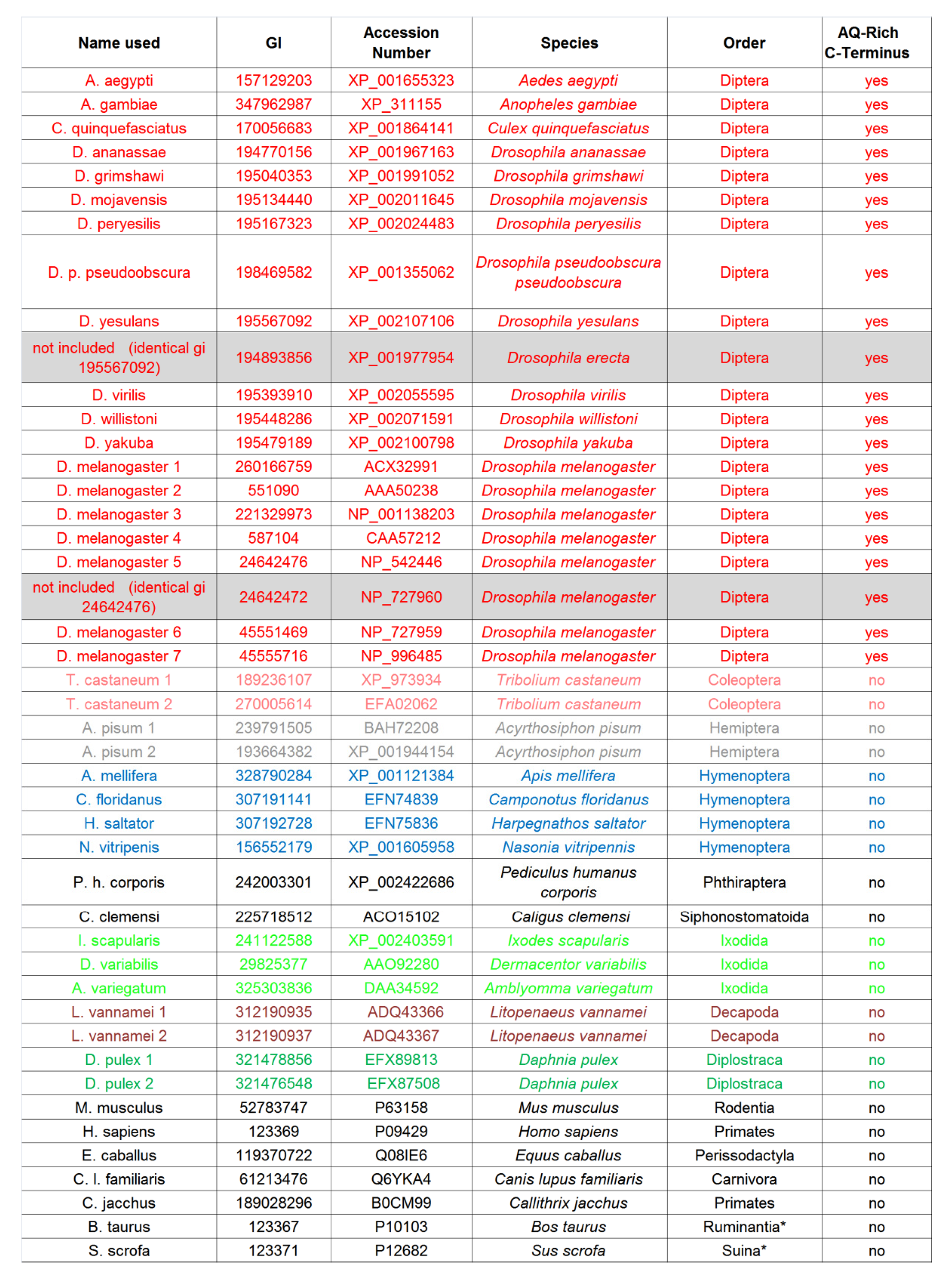

Supplement: Table S1 — Description of Orders containing the AQ-rich C-terminal domain. HMGB1-containing organisms were separated by their Orders and analyzed for the presence of an AQ-rich domain in their C-terminus. Note that among the insects, the presence of an AQ-rich domain in the C-terminus of HMGB1 proteins is a peculiarity of the dipterans. Such a domain does not exist in mammals either. The different Orders are represented by the different colors. Asterisks indicate infraorder or suborder, when Orders were not found in the NCBI taxonomy database. (TIF) [file pone.0040192.s003.tif]
